# Insulin Receptor-Overexpressing β-Cells Ameliorate Hyperglycemia in Diabetic Rats through Wnt Signaling Activation

**DOI:** 10.1371/journal.pone.0067802

**Published:** 2013-07-09

**Authors:** Mi-Hyun Kim, Seung-Hyun Hong, Moon-Kyu Lee

**Affiliations:** 1 Division of Endocrinology and Metabolism, Samsung Biomedical Research Institute, Seoul, Korea; 2 Bionano Research Center, Korea Research Institute of Bioscience and Biotechnology (KRIBB), Daejeon, Korea; 3 Division of Endocrinology and Metabolism, Department of Medicine, Samsung Medical Center, Sungkyunkwan University School of Medicine, Seoul, Korea; Broad Institute of Harvard and MIT, United States of America

## Abstract

To investigate the therapeutic efficacy and mechanism of β-cells with insulin receptor (IR) overexpression on diabetes mellitus (DM), rat insulinoma (INS-1) cells were engineered to stably express human insulin receptor (INS-IR cells), and subsequently transplanted into streptozotocin- induced diabetic rats. Compared with INS-1 cells, INS-IR cells showed improved β-cell function, including the increase in glucose utilization, calcium mobilization, and insulin secretion, and exhibited a higher rate of cell proliferation, and maintained lower levels of blood glucose in diabetic rats. These results were attributed to the increase of β-catenin/PPARγ complex bindings to peroxisome proliferator response elements in rat glucokinase (GK) promoter and the prolongation of S-phase of cell cycle by cyclin D1. These events resulted from more rapid and higher phosphorylation levels of insulin-signaling intermediates, including insulin receptor substrate (IRS)-1/IRS-2/phosphotylinositol 3 kinase/v-akt murine thymoma viral oncogene homolog (AKT) 1, and the consequent enhancement of β-catenin nuclear translocation and Wnt responsive genes including GK and cyclin D1. Indeed, the higher functionality and proliferation shown in INS-IR cells were offset by β-catenin, cyclin D1, GK, AKT1, and IRS-2 gene depletion. In addition, the promotion of cell proliferation and insulin secretion by Wnt signaling activation was shown by 100 nM insulin treatment, and to a similar degree, was shown in INS-IR cells. In this regard, this study suggests that transferring INS-IR cells into diabetic animals is an effective and feasible DM treatment. Accordingly, the method might be a promising alternative strategy for treatment of DM given the adverse effects of insulin among patients, including the increased risk of modest weight gain and hypoglycemia. Additionally, this study demonstrates that the novel mechanism of cross-talk between insulin and Wnt signaling plays a primary role in the higher therapeutic efficacy of IR-overexpressing β-cells.

## Introduction

A cure for type 1 diabetes and some cases of type 2 diabetes would require the means to replace the functions of deficient insulin-secreting β-cells to regulate abnormal levels of blood glucose. Most studies have focused on islet or β-cell transplantation for the treatment of diabetes. However, the limited supply of islets/β-cells is always an obstacle to treatment procedures [1]. Thus cell therapy with gene manipulation that confers β-cells with higher proliferative ability and functionality has emerged as an alternative and desirable method for the treatment of diabetes [2].

Recently, variants of transcription factor 7-like 2 (TCF7L2), a component of Wnt/β-catenin signaling, have been shown to be involved in β-cell dysfunction and the pathogenesis of type 2 diabetes [3]. In addition, a link between glucose sensing, cell proliferation and Wnt/β-catenin signaling has been reported in macrophages, β-cells, and colonic cells [4]–[6]. Glucokinase (GK) plays a glucose-sensing role in pancreatic β-cells and hepatocytes, and functions in the glucose-dependent modulation of insulin secretion [7]. In addition, previous studies have revealed that β-catenin activates GK promoter activity in the presence of the peroxisome proliferator-activated receptor *(*PPAR)γ [5], and that the cyclin D1 promoter contains consensus TCF7L2 binding sites. Furthermore, a dominant–negative form of TCF7L2 causes cells to arrest in the G1-phase of the cell cycle. These results strongly suggest that Wnt signaling could regulate β-cell functionality and proliferation.

Interestingly, insulin treatment in intestinal L-cells enhances nuclear translocation and binding of β-catenin to TCF7L2 through the phosphatidylinositol 3 kinase (PI3K)-dependent inhibition of glycogen synthase kinase 3β (GSK3β), an inhibitor of β-catenin [8]. Furthermore, cross-talk between insulin and Wnt signaling pathways has been revealed to exist in a variety of cells, including preadipocytes, skeletal muscle cells, and intestinal cells [9]–[11], but not yet in β-cells. Generally, early intensive insulin therapy controls hyperglycemia and delays β-cell damage and restores β-cell function in type 2 diabetic patients. However, insulin therapy can induce adverse effects, however, including modest weight gain and hypoglycemia. In this regard, the replacement with β-cells with IR overexpression could be a promising tool in the treatment of diabetes mellitus (DM), as long as the insulin receptor (IR)-overexpressing β-cells show similar functions and abilities as insulin through Wnt signaling.

Accordingly, we hypothesized that the overexpression of IR in β-cells could amplify PI3K/v-akt murine thymoma viral oncogene homolog (AKT), capable of inhibiting GSK3β-dependent β-catenin degradation [12], which could activate Wnt responsive genes cyclin D1 and GK [13], and subsequently promote β-cell functionality.To investigate the therapeutic efficacy of β-cells with IR manipulation, a plasmid containing human IR was stably introduced into rat β-cell line INS-1 cells (namely, INS-IR cells). In the INS-IR cells, the role of cross-talk between insulin and Wnt signaling pathways in improving the functionality of β-cells was investigated with a transient β-catenin small interfering RNA (siRNA).

## Materials and Methods

### Animals and Transplantation

Eight-week-old male Sprague-Dawley rats (Harlan, Indianapolis, IN) were housed at a constant temperature (23.5±2.0°C) and humidity (50±5%), with a 12∶12-hour light/dark cycle. The animals had free access to water and food (Purina Formula, St. Louis, MO). The rats were injected intraperitoneally with a 40 mg/kg solution of streptozotocin (STZ, 0.05 mM, pH 4.5, which was freshly dissolved in citric acid and sodium citrate) for 5 consecutive days. Once nonimplanted mice reached blood glucose levels >300 mg/dl, the left kidney was exposed through a small incision in the flank. A small channel was made under the renal subcapsular space of the kidney with a 23-gauge needle, through which 2×10^5^ of INS-1 and INS-IR cells were transplanted into the subcapsular space of the kidney in the diabetic rat. The non-fasting blood glucose levels were monitored at 09∶00 every day following transplantation. Blood was drawn from the tail for determination of glucose levels (AccuCheck Active, Roche Applied Science). Blood glucose levels were monitored daily until killed. The rat were anesthetized and then killed by cervical dislocation. Serum was collected for insulin levels analyses using a rat insulin radioimmunoassay kit (Linco Research, St. Louis, MO) and kidneys were harvested for immunohistochemical analyses.

Animal studies were conducted with the approval (SBRI No. C-A9-228-1) of and in accordance with the guidelines of the Animal Care and Use Committee of the Samsung Biomedical Research Institute, which complies with national and international laws and policies (National Institutes of Health Guide for Care and Use of Laboratory Animals, 7^th^ edition, 1996).

### Cell Culture

The INS-1 rat insulinoma cells were maintained in RPMI 1640 (Gibco/Invitrogen, Burlington, ON, CA) supplemented with 10% FBS, 2 mM L-glutamine, 100 U/ml penicillin, 100 μg/ml streptomycin, 1 mM sodium pyruvate, and 50 μM 2-mercaptoethanol.

### Stable INS-IR Cells

Plasmid pCMV6 containing a neomycin selection cassette harboring human IR cDNA (pCMV6-hIR) was used. The pCMV6-hIR or empty pCMV6 (as control) was transfected using Effectene (Qiagene, Chatsworth, CA) into INS-1 cells. After 24–48 h of transfection, 1.0×10^2^ cells were plated onto 10 cm plates containing selection medium with 600 μg/ml neomycin/G418 (Sigma-Aldrich, St. Louis, MO). After 2–4 weeks, G418 resistant colonies were isolated using a colony separator (Sigma-Aldrich), and seeded into one well of a 96-well plate containing selection medium. These 4 clones were further screened for the positive clones through glucose-stimulated insulin secretion (GSIS) response. Based on insulin secretion response, Clone 4 was selected as the primary clone for INS-IR cell (Figure S1 in File S1). All the further studies were performed on the primary clone, Clone 4.

### Insulin Treatment

Insulin and cycloheximide were from Sigma Chemical Co. INS-1 cells were transferred to a glucose- and serum-free RPMI 1640 medium for 24 h and then were exposed to insulin (0.1–100 nM) in serum-free RPMI-1640 medium for 4 h. To abolish the reflection of *de novo* synthesis of insulin or endogenous autocrine insulin signaling in INS-1 cells, cycloheximide (10 μg/ml in ethanol), an inhibitor of translational elongation in eukaryotic organism, was added 30 min prior to insulin treatment [14]. After insulin treatment, insulin secretion was measured by the equal method of GSIS.

### TOPflash/FOPflash Transfection

In cDNA TOPflash and FOPflash plasmids (Millipore, Billerica, MA), expression of a luciferase (LUC) reporter gene is driven by a minimum TK promoter, fused with three copies of the TCF7L2 binding sites and the mutant TCF7L2 binding site. Approximately 5×10^4^ INS-IR cells were seeded in wells of a 24-well plate for 24 h, and then transfected with 100 ng of TOPflash or FOPflash using Lipofectamine 2000 (Invitrogen) for 24 h. After transfection, the cells were exposed to 33.3 mM glucose for measuring insulin secretion. The activity of TOPflash and FOPflash was analyzed with LUC reporter assay (Promega, Madison, WI).

### Immunoprecipitation

For immunoprecipitation (IP), cells were lysed by lysis buffer (20 mM Tris-HCl; pH 7.5, 150 mM NaCl, 1 mM EDTA, 1vmM EGTA, 1% Triton X-100, 2.5 mM sodium pyrophosphate, 1 mM β-glycerol phosphate) containing NaF, phenylmethanesulfonyl fluoride (PMSF), and Na3VO4, and were centrifuged at 12,000 g for 10 min at 4°C. Proteins were immunoprecipitated with primary antibodies for 2 h at 4°C and reacted with protein A-agarose for 1 h. The immunoprecipitates were washed with lysis buffer A and solubilized in a sodium dodecyl sulfate (SDS) sample buffer (63.5 mM Tris-HCl; pH 6.8, 2% w/v SDS, 10% glycerol, 50 mM dithiothreitol (DTT), 0.01% w/v bromophenol blue). The proteins were separated by 10% SDS-polyacrylamide gel electrophoresis (SDS-PAGE), transferred onto nitrocellulose membranes, and visualized with other primary antibodies (1∶1000) followed by horseradish peroxidase-labeled secondary antibodies (1∶5000 dilution).

### Chromatin Immunoprecipitation

Crosslinking of DNA with histones was performed by adding formaldehyde to culture medium (1% final concentration) for 10 min at 37°C. After washing, the cells were scraped, centrifuged, and lysed in chromatin immunoprecipitation (ChIP) lysis buffer (Upstate Biotechnology, Lake Placid, NY). The lysates were sonicated to shear DNA to lengths between 200 and 1000 bp. The sheared chromatin (supernatant) was diluted with ChIP dilution buffer (Upstate Biotechnology) and pre-cleared with salmon sperm DNA/Protein A-agarose 50% slurry (agitation for 30 min at 4°C), and subsequently transferred and divided into two fractions (IP and NoAb). A β-catenin antibody (Santa Cruz Biotechnology, Santa Cruz, CA) was added to the IP sample, and the fraction without antibody (NoAb) was used for quantification of total chromatin. Both fractions were incubated overnight at 4°C on a rotator, and then incubated for 1 h at 4°C with 60 µl of salmon sperm DNA/Protein A-agarose slurry. The A-agarose/antibody/histone pellets were serially washed once with 1 ml low salt, high salt, and LiCl wash buffer, and twice with 1xTE buffer (10 mM Tris base, 1 mM EDTA). To elute any histones caught by the antibody, the pellets were resuspended in 250 µl SDS elution buffer (1% SDS, 0.1 M NaHCO_3_) for 15 min at room temperature. In order to reverse crosslink histones and DNA, the eluted supernatants were added to 20 µl 5 M NaCl at 65°C for 4 h, and then to 10 µl 0.5 M EDTA, 20 µl 1M Tris-HCl (pH 6.5), and 2 µl 10 mg/ml proteinase K at 45°C for 1 h. DNA isolation was performed by phenol/chloroform extraction and ethanol precipitation. The region and sequence of peroxisome proliferator response elements (PPRE) in rat-specific β- cell GK promoters (NC_005113.2, Chr14q21) was quoted in a previous study [15].

### Glucose-Stimulated Insulin Secretion (GSIS)

Cells were preincubated for 2 h at 37°C in Krebs-Ringer-bicarbonate-HEPES (KRB) buffer plus 0.5% BSA for glucose deprivation condition, and then washed twice with KRB buffer plus 0.5% BSA. Next, cells were incubated for an additional 60 min at 37°C in 1 ml KRB buffer containing 3 mM and 33.3 mM glucose. After glucose stimulation, the supernatant were collected and determined insulin release by using a rat insulin radioimmunoassay kit (Linco Research, St. Louis, MO).

### Perifusion

Cells were preincubated for 30 min at 37°C in KRB buffer containing 3.3 mM glucose. These cells were put onto a column between two layers of filter (Millipore Corporation, Bedford, MA) and perifused at a flow rate of 0.3 ml/min for 30 min in KRB buffer containing 3.3 mM glucose. This was followed by glucose stimulation (33.3 mM) for 60 min. Insulin (collected per interval of 2–3 min) was quantified by rat insulin RIA (Linco Research).

### Glucose Transport

Cells were serum-starved for 3 h, and washed three times with HEPES buffer containing 1% bovine serum albumin (BSA), 10 nM HEPES, 131.2 mM NaCl, 4.7 mM KCl, 1.2 mM MgSO_4_, 2.5 mM CaCl_2_, and 2.5 mM NaH_2_PO_4_, pH 7.6. The cells were incubated with the same HEPES buffer for 1 h at 37°C. Subsequently, 0.2 uCi of 2-[^3^H] glucose (final concentration of 33 mM) was added for 5 min. The cells were washed with cold phosphate buffered saline (PBS) and solubilized with 0.1 N NaOH. The radioactivity incorporated into the cells was measured by liquid scintillation counting.

### Measurement of Intracellular ATP

After 33.3 mM glucose stimulation, cells were harvested and 2.5% trichloroacetic acid (TCA) was added to extract ATP. The supernatant containing TCA was neutralized with Tris-acetate (pH 7.75). Measurement of ATP content was performed by a luciferin/LUC method (Promega) and determined using a Wallac Trilux 1450 Microbeta liquid scintillation counter (Wallac, Turku, Finland).

### Measurement of Intracellular Calcium Concentration ([Ca^2+^]_i_)

Cells were loaded with Fura-2-acetoxylmethyl ester (4 M) in KRB buffer for 30 min, and then washed twice with KRB and incubated for an additional 30 min at 37°C. Dishes were placed into a heated chamber mounted on the stage of an inverted fluorescence microscope and perifused with KRB plus 3.3 mM glucose at a rate of 1.5 mL/minat out with fresh solution. Baseline was established for at least 6 min before stimulation (33.3 mM). The level of [Ca^2+^]_i_ was assessed using a microfluorometric system consisting of an IX-70 inverted fluorescence microscope (Olympus, Tokyo, Japan) with a dry-type fluorescence objective (×40, NA 0.85), a type R 1527 photomultiplier tube (Hamamatsu, Shizuoka, Japan) and a Deltascan illuminator (Photon Technology International, Lawrenceville, NJ). Light was provided by a 75-W xenon lamp (Ushino, Tokyo, Japan). A chopper wheel alternated the light path to monochromators (340 and 380 mM) with a frequency of 5 Hz, and the intensity of the emitted light was measured at 510 nm. The ratio of the fluorescence emissions at 340 and 380 nm excitation was taken as a measure of [Ca^2+^]_i_.

### Cell Growth and Doubling Time

Cells were grown for 72 h in culture medium after serum-free starvation. To estimate cell volume growth, the doubling time (DT) was calculated using the Schwartz equation:


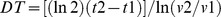
, where t is time and v is volume.

### Cell Cycle Analysis by Flow Cytometry

Cells were harvested, resuspended in PBS containing 1% FBS, and then fixed with 70% ethanol. Cells were resuspended again in PBS containing 30 μg/ml propidium iodide and 250 mg/ml RNase A, and further incubated at 4°C for 1 h before analysis with a FACSCalibur laser-based flow cytometer (BD Biosciences, Franklin Lakes, NJ). The cell cycle phase distribution was analyzed with FLOWJO software (Tree Star, Ashland, OR.).

### BrdU Incorporation

Two days before the procedure, cells were seeded onto 25-mm glass coverslips at a density of 2×106 cells/ml, and 5-bromo-2′-deoxyuridine (BrdU, 10 μM) was added to the medium. BrdU was detected with immunohistochemistry utilizing an anti-BrdU monoclonal antibody according to the manufacturer's recommendations (Roche Diagnostics, Indianapolis, IN).

### Apoptosis Assay

Cells were cultured for 4–6 h in serum-free medium, and then treated with 33.3 mM glucose, 0.5 mM oleic acid, 100 uM 30% hydrogen peroxide (H_2_O_2_), and 10 ng/ml tumor necrosis factor-alpha (TNF-α), as well as the siRNA of IRS-2, AKT1, β-catenin, and cyclin D1. Apoptotic cells were labeled with an annexin V-Fluos kit and confirmed by flow cytometry.

### Immunofluorescence (IF) and Immunohistochemistry (IHC)

Immunofluorescent cells were incubated with primary β-catenin antibodies (1∶200) followed by secondary antibodies (1∶1000) labeled with fluorescein isothiocyanate (Invitrogen). Nuclei were stained with Hoechst 33258 (0.5 mM in PBS; Sigma-Aldrich). Isolated rat kidney tissues for IHC were fixed with 10% formalin, embedded in paraffin, mounted on slides, deparaffinized in xylene and ethanol, and blocked with 10% normal horse serum. Tissues were incubated with primary antibodies (1∶200 dilution) overnight at 4°C followed by the addition of biotinylated anti-rabbit IgG and then avidin-biotin-peroxidase complex (Vector Laboratories, Burlingame, CA). Nuclei were stained with hematoxyline (Sigma-Aldrich). The BrdU positive kidney cells were detected using a cell proliferation kit (Amersham Pharmacia Biotech, Piscataway, NJ) according to the manufacturer's instructions. Images were captured on an Eclipse TE 200 confocal microscope (Nikon, Tokyo, Japan).

### Western Blotting

Cells were lysed with a buffer (20 mM Tris-HCl, 150 mM NaCl, 1 mM EDTA, 1% Triton X-100) containing proteinase inhibitors (1 mM aprotinin, 1 mM leupeptin, 1 mM PMSF) and protease inhibitors (1 mM NaOV_3_, 1 mM NaF) at 4°C. The lysates were cleared by centrifugation at 13,000 rpm for 15 min, and the supernatant protein content was quantified. Twenty micrograms of protein were separated by 10% sodium dodecyl sulfate-polyacrylamide gel electrophoresis (SDS-PAGE) and transferred onto nitrocellulose membranes. Proteins were visualized with primary antibodies, followed by horseradish peroxidase-labeled anti-goat, rabbit, and/or mouse Ig (1∶5000 dilution; Santa Cruz Biotechnology).

### RNA interference

Small interfering RNA (siRNA) against rat β-catenin, GK, AKT1, IRS-2, Cyclin D1 and scrambled RNA (as control) were purchased from Dharmacon Research (Lafayette, CO). INS-1 cells (5×10^5^ cells) were seeded in 6-well plates for 24 h and then transfected with 50 nM siRNAs using Lipofectamine 2000 (Invitrogen) for 48 h. The magnitude of knockdown of gene was quantified by Image J program (Figure S3–S6 in File S1).).

### Microarray Analysis

Each sample, 500 ng of RNA were linearly amplified and fluorescently labeled with either Cy3-CTP with the Agilent Low RNA Input Fluorescent Linear Amplification Kit (Agilent Technologies, Amstelveen, The Netherlands). Equal amounts (1 µg) of Cy3-CTP labeled samples were hybridized to Agilent 4×44 K Whole Rat Genome arrays (Agilent Technologies) according to the manufacturer's instructions. Microarrays were scanned using an Agilent DNA Microarray Scanner, and scans were quantified using Agilent Feature Extraction software (version 8.5.1). Raw expression data generated by the Feature Extraction software. Target sequence signals were normalized using Per Spot and Per Chip: Intensity Dependent (Lowess) Normalization in Agilent's GeneSpring Software. After normalization, the separate intensity channels were extracted from the ratio measurements and combined to the intensities of the reference. Biological processes were analyzed via Protein Analysis Through Evolutionary Relationships (PANTHER; http://www.pantherdb.org). The microarray data were deposited in ArrayExpress (Access number: E-MEXP-3836; Repository name: ArrayExpress EBI) and listed in Table 1.

**Table 1:** Gene expression profiles altered by IR overexpression.

Gene bank ID	Gene symbol	Gene description	Fold change
**Insulin signaling-related genes**			
NM_013005	Pik3r1	phosphatidylinositol 3-kinase, regulatory subunit, polypeptide 1	1.50
BE118080	Irs2	Insulin receptor substrate 2	2.64
NM_053483	Kpna2	karyopherin (importin) alpha 2	1.59
**Wnt signaling-related genes**			
NM_021266	Fzd1	frizzled homolog 1 (Drosophila)	2.65
NM_153474	Fzd3	frizzled homolog 3 (Drosophila)	2.62
XM_232466	Lrp6	low density lipoprotein receptor-related protein 6	0.82
AF486617	Ctnnb1	catenin (cadherin associated protein), beta 1	0.94
NM_053369	Tcf4	transcription factor 4	1.03
NM_001024252	Pcaf	p300/CBP-associated factor	2.07
NM_012499	Apc	adenomatosis polyposis coli	0.53
NM_024355	Axin2	axin2	0.78
NM_024405	Axin1	axin 1	0.61
NM_031617	Csnk1e	casein kinase 1, epsilon	0.87
NM_053615	Csnk1a1	casein kinase 1, alpha 1	0.80

The fold changes were represented with the mean gene expression ratios from microarray experiments comparing INS-1cells with INS-IR cells.

### Total RNA Extraction and Real-Time PCR

Total RNA was isolated and reverse-transcribed using TRIzol reagent and a reverse transcriptase kit (Invitrogen, Carlsbad, CA) according to the manufacturer's instructions. For the relative quantification of gene expression, real-time PCR was conducted using a Biosystem 7500 real-time PCR system (Applied Biosystems, Foster City, CA). Five hundred nanograms of cDNA were amplified by a 25 μl SYBR Green PCR master mix (Applied Biosystems) and specific primer pairs (Table 2). The relative expression levels of target genes were calculated as 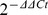, where 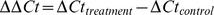 and 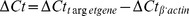.

**Table 2:** Sequences of primers used in RT-PCR.

Gene name	Forward Primer	Reverse Primer	Accession number	Amplicon size (bp)
GSK3β	CAGTGGTGTGGATCAGTTGG	AATTTGCTCCCTTGTTGGTG	NM_032080.1	64
β-catenin	CATATGCGGCTGCTGTTCTA	CCGAAAGCCGTTTCTTGTAG	NM_053357.2	69
TCF7L2	GTCCACCCACTCACACCTCT	TTCCTGTTTTGGGGTCTACG	NM_001191052.1	100
Cyclin D1	ATGCTAGAGGTCTGCGAGGA	GGCTCCAGAGACAAGAAACG	NM_171992.4	89
IRS-2	TATACCGAGATGGCCTTTGG	CCATGAGACTTAGCCGCTTC	NM_001168633.1	121
IRS-1	GGCTGACTCCAAGAACAAGC	CTTGTTCAGCCTCGCTATCC	NM_012969.1	86
Insulin	GCCTTTGTGAACCAACACCT	CGGGTCTTGGGTGTGTAGAA	NM_001185097.1	95
GLUT2	GGACAAACTCGGAAGGATCA	AATTTGGAACACCCCATCAA	NM_012879.2	84
AKT1	TCAGGTTCACCCAGTGACAA	TCTCCTTCACCAGGATCACC	NM_033230.1	145
GK	TGAGGGACAGAGTTACCTGTTG	GGGGATGGCTTTTGAGTAATG	NC_005113.2	48

The sets of primer sequences were designed by Primer Express Software v2.0 (ABI, USA) and verified with a basic local alignment search tool (BLAST) provided by the National Center for Biotechnology Information (NCBI).

### Statistical Analyses

Data were expressed as the mean ± SEM, and were analyzed using t-tests or via ANOVA with Duncan's multiple range test using SAS Proprietary Software Release 8.2 (SAS Institute, Cary, NC).The data with dissimilar superscript letters (a, b, c, etc.) and/or * were analyzed by ANOVA post-hoc Duncan's multiple range test and/or Student t-test, respectively. Data with p-values <0.05 were considered statistically significant.

## Results

### Improvement of β-cell functions by IR overexpression

**Figure 1:**
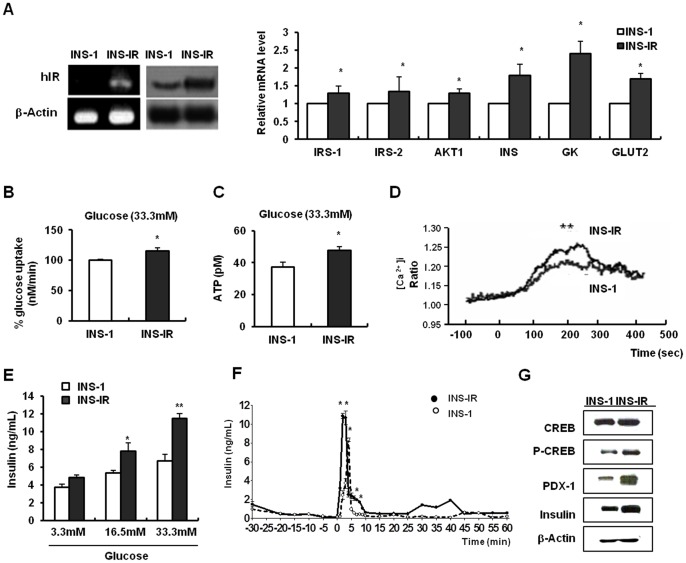
IR overexpression improves β-cell functions. The plasmid pCMV6 containing the human IR cDNA was stably transfected into INS-1 NS-IR cells). *A*, Left panel: Comparison of hIR band by using RT-PCR and the western blot method. Right cells (I panel: Relative mRNA levels of β-cell markers and insulin signaling intermediators using real time PCR. *B*, Radio-labeled glucose incorporation into INS-1 and INS-IR cells in response to 33.3 mM glucose stimulus. *C*, Intracellular ATP concentration using a luciferin/LUC method. *D*, Fura-2 AM staining Ca^2+^ influx into cells using a microfluorometric system. *E*, Insulin concentration in media in response to glucose stimulus. *F*, First-phase and second-phase insulin release pattern. Insulin secretion was tested by perfusion with KRBB containing 33.3 mM glucose. *G*, Protein levels of CREB/PDX-1/insulin using the western blot method. Values are mean ± SE of three independent experiments, each performed in triplicate. *P<0.05, **P<0.001 INS-1 vs. INS-IR cells (Student t-test).

INS-IR cells showed a higher expression of the hIR gene at mRNA and protein levels than INS-1 cells. Also, INS-IR cells displayed a higher expression of β-cell marker genes including insulin, GK, and glucose transporter 2 (GLUT2) and the insulin signaling pathway intermediators such as insulin receptor substrate (IRS)-1, IRS-2 and AKT1, than the INS-1 cells (P<0.05, Figure 1A). The cells showed improvement in various parameters of β-cell functions such as glucose uptake, intracellular ATP content, Ca^2+^ mobilization, insulin secretory ability, and gene expression in comparison to INS-1 cells (Figure 1B-G). At the higher glucose concentration (33.3 mM), INS-IR cells showed increased glucose uptake and intracellular ATP levels in comparison to INS-1 cells (P<0.05, Figures 1B and C). Subsequently, L-type Ca^2+^ channels were opened due to membrane depolarization. In INS-1 and INS-IR cells, the baseline [Ca^2+^]i varied between 1.0 and 1.1. In the presence of 33.3 mM stimuli, however, [Ca^2+^]i rose more rapidly in response to glucose in INS-IR cells (0.275±0.018) than in INS-1 cells (0.199±0.029) (P<0.05, Figure 1D). The [Ca^2+^]i was expressed as 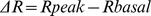; that is, the fluorescence level at the peak obtained with high glucose (Rpeak) and the basal level of fluorescence (Rbasal). An increase in Ca^2+^ modulation induces the exocytosis of insulin granules. The secretion of insulin was increased 1.32±0.09-fold in INS-IR cells over INS-1 cells in response to 33.3 mM glucose (P<0.05, Figure 1E). Moreover, perifusion was performed to observe the dynamic insulin response after the glucose challenge. INS-IR cells showed significantly increased first-phase and second-phase insulin release (first peak: 2.5-fold, second peak: 1.7-fold), as compared with INS-1 cells (P<0.05, Figure 1F). Increased insulin secretion or perifusion resulted from the increase in calcium-dependent protein kinase, cAMP response element binding protein (CREB), its downstream gene pancreatic and duodenal homeobox-1 (PDX-1), and a subsequent insulin gene (Figure 1G).

Also, glucose uptake, ATP content, perifusion, and insulin secretion were determined in response to 16.7 mM glucose stimulus (Figure 1E and Figure S2A–C in File S1).) but these parameters, except for insulin secretion, were not significantly different between INS-1 and INS-IR cells. Moreover, low doses of STZ 40 mg/kg, i.p. to rat for five consecutive days induced the higher blood glucose levels (>400 mg/dl) in our study. Considering these points, we exposed INS-1 or INS-IR cells to the 33.3 mM glucose for following mechanism study. Collectively, IR overexpression improves β-cell functionality.

### Improved β-cell functionality occurs through Wnt signaling and GK transcription

**Figure 2:**
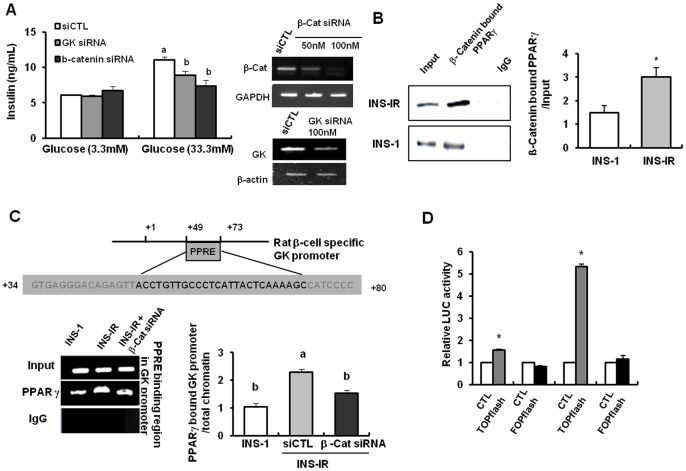
Improvement of β-cell function occurs through β-catenin-dependent GK transcription. The plasmid pCMV6 containing the human IR cDNA was stably transfected into INS-1 cells (INS-IR cells). *A*, Left panel: Insulin secretion after transfection of β-catenin and GK siRNA into INS-IR cells. Right panel: The magnitude of the knockdown of each gene after transfection of β-catenin and GK siRNA. *B*, Interaction of PPARγ with β-catenin using IP analysis. C, The binding of PPARγ/β-catenin complex to PPRE in β-cell-specific GK promoters using ChIP analysis. The GK-PPRE is located in the region between +47 and +68 bp (expressed in red letters). *D*, Relative LUC activity after transfection of TOP- and/or FOPflash into INS-IR cells. Transfected INS-IR cells were stimulated by 3.3 or 33.3 mM glucose. Values are mean ± SE of three independent experiments, each performed in triplicate. ^a,b,c^ Mean values with dissimilar superscript letters were significantly different between each group (ANOVA post-hoc Duncan's multiple range test; P<0.05). *P<0.05, CTL plasmid vs. TOP/FOP flash plasmid or INS-1 vs. INS-IR cells (Student t-test).

**Figure 3:**
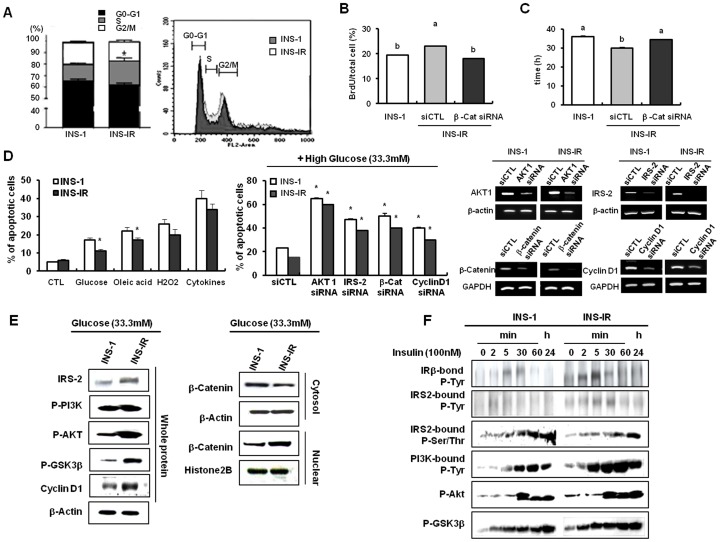
IR overxpression enhances β-cell proliferation via β-catenin-dependent cyclin D1 transcription. The plasmid pCMV6 containing the human IR cDNA was stably transfected into INS-1 cells (INS-IR). *A*, Cell cycle analysis using FACS. *B*, BrdU incorporation into INS-IR and/or INS-IR cells. *C*, Doubling time in INS-1 and/or INS-IR cells. Doubling time was calculated using the Schwartz equation. *D*, Left panel: Apoptosis rate by 0.5 mM oleic acid (O.A), 33.3 mM glucose, 100 M H_2_O_2_, and cytokine TNFα (10 ng/ml) and IFNγ (20 ng/ml). Middle panel: Glucose-induced apoptosis rate after transfection of β-catenin, cyclin D1, IRS-2, and AKT1 siRNAs into INS-1 and/or INS-IR cells. Apoptosis was analyzed by annexin V-Fluos kit and confirmed by flow cytometry. Right panel: The magnitude of the knockdown of each gene after transfection of β-catenin, cyclin D1, IRS-2, and AKT1 siRNAs into INS-1 and/or INS-IR cells. *E*, Protein levels of genes involved in insulin-signaling and Wnt- signaling pathways using the western blot method. *F*, Insulin signaling in INS-1 and/or INS-IR cells treated with 100 nM insulin for 2, 5, 30, and 60 min, and 24 h. Values are mean ± SE of three independent experiments, each performed in triplicate. ^a,b,c^ Mean values with dissimilar superscript letters were significantly different between each group (ANOVA post-hoc Duncan's multiple range test; P<0.05). *p<0.05, INS-1 vs. INS-IR cells or CTL siRNA vs. Each gene siRNA (Student t-test).

The transfection of β-catenin siRNA into INS-IR cells diminished the effects of IR overexpression on GSIS (33.3 mM, P<0.05, Figures 2A and B), compared to scrambled siRNA. These results might be attributed to the reduced transcriptional activity of GK, a glucose sensor in β-cells, by blocking Wnt signaling pathways. Indeed, depletion of GK with siRNA reduced GSIS in INS-IR cells as shown in β-catenin depletion (P<0.05, Figure 2A and Figure S1 in File S1). and Figure S2 in File S1).). Moreover, in IP and ChIP analyses, INS-IR cells showed significantly increased levels of the PPARγ that binds to β-catenin and the binding of β-catenin/PPARγ complex to PPRE (+47∼+68 bp) in rat β-cell GK promoters in comparison to the INS-1 cells (P<0.05, Figure 2B and C). Taken together, our data suggest that an increase in GSIS by IR overexpression was obtained by augmenting the binding of β-catenin/PPARγ complex to PPRE in rat GK promoters, which is a process that depends on the enhancement of β-catenin stabilization and nuclear translocation by IR overexpression. Indeed, IR overexpression activated the TOPflash reporter, but not FOPflash reporter, and thus these findings implied the enhancement of the transcriptional activity of β-catenin-TCF/LFE (P<0.05, Figure 2D). This TOPflash activation was probably mediated by engaging endogenous β-catenin because IR overexpression increased levels of nuclear translocation of β-catenin (Figure 3E). These results were confirmed with microarray data showing that most of the genes involved in Wnt signaling pathways, such as Frizzled homolog (Fzd) 1 and 5, LRP5L,and PCAF and insulin-signaling intermediates such as PIK3 and IRS2, were upregulated in INS-IR cells compared to INS-1 cells (Table 1). Also, Wnt signaling inhibitors, APC, Axin2 and CSNK1 were downregulatd in INS-IR cells compared to INS-1 cells (Table 1).

### Enhanced β-cell proliferation results from β-catenin-dependent cyclin D1 transcription

Along with the improved functionality of β-cells, INS-IR cells also showed increased proliferation rate. First, the S-phase of the cell cycle in which DNA is replicated was more prolonged in INS-IR cells (G0/G1 60%, S 23% and G2/M 17%) than INS-1 cells (G0/G1 68%, S 12% and G2/M 20%) (P<0.05, Figure 3A). These results are similarly with a study comparing cell cycle progression of rodent insulinoma cell lines including INS-1 with primary rodent β-cells, which showed that the two rat lines (RINm5F and INS1) showed a decreased G0/G1 population (70–80%) and an increased proportion (24–46%) of cells actively engaged in the cell cycle (S and G2/M), unlike primary rat islet cells, which are arrested in G0/G1 (96%) [16].

Second, the BrdU incorporation assay showed that the number of BrdU positive cells was greater in INS-IR cells (23.23±1.419) than those in INS-1 cells (18.11±0.765) (P<0.05, Figure 3B), and third, the doubling time of cells was shortened in INS-IR cells in comparison to INS-1 cells (P<0.05, Figure 3C). However, the number of BrdU positive cells was reduced by transfecting β-catenin siRNA (P<0.05, Figure 3B) and the doubling time of INS-IR cells was elongated by transfecting β-catenin siRNA (P<0.05, Figure 3C). We also investigated the rate of apoptosis using an annexin V assay. The 24-hour exposure to glucose and oleic acid modestly increased the rate of apoptosis. However, the rate of apoptosis was reduced in INS-IR cells in comparison to INS-1 cells (P<0.05, Figure 3D). Furthermore, depletion of Wnt signaling effectors, such as β-catenin and cyclin D1, as well as insulin-signaling molecules and potent anti-apoptotic materials, AKT1 and IRS-2, amplified the rate of glucose-induced apoptosis in INS-IR cells (P<0.05, Figure 3D and Figure S3–S6 in File S1).). These results suggest that higher proliferative ability by IR manipulation might be associated with cross-talk between insulin and Wnt signaling pathways.

Indeed, INS-IR cells showed increased IRS-2 expression and increased phosphorylation of PI3K and AKT in comparison to INS-1 cells in the presence of 33.3 mM glucose (Figure 3E). These events resulted in phosphorylation of GSK3β at serine residue 9, thereby inducing nuclear translocation of β-catenin and the increase of cyclin D1 expression in INS-IR cells (Figure 3E). Additionally, IR overexpression rapidly transmitted insulin signaling to Wnt signaling (Figure 3F). In IP analysis with cells stimulated by 100 nM insulin, maximal tyrosine phosphorylation of IRβ, IRS-1, and PI3K, as well as serine 9 phosphorylation of GSK3β, were detected more rapidly in INS-IR cells than in INS-1 cells.

### Insulin activates Wnt signaling similarly to IR overexpression

**Figure 4:**
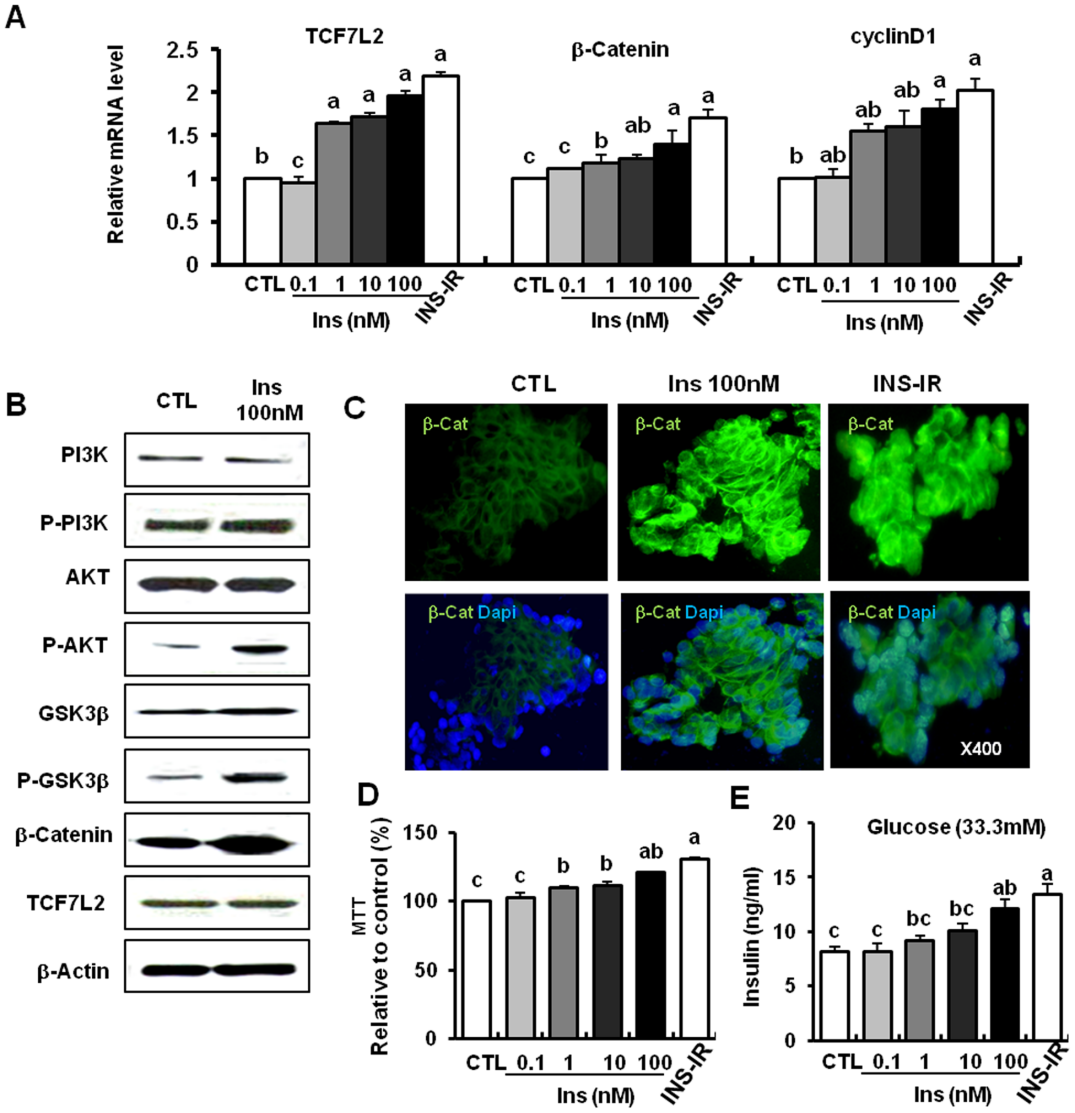
Insulin activates Wnt signaling similarly to IR overexpression. *A*, Relative mRNA levels of Wnt intermediates (β-catenin, TCF7L2, cyclin D1) using real time PCR. *B*, Protein levels of genes involved in insulin and Wnt signaling in the presence of 100 nM insulin using the western blot method. *C*, Nuclear translocation of β-catenin by 100 nM insulin or IR overexpression (the green color represents β-catenin and the blue color represents DAPI); original magnification, ×400. *D*, MTT assay. The cell proliferation index is expressed as a percentage of the mean value measured in the untreated controls. *E*, Insulin secretion in response to glucose (33.3 mM) in the presence of 100 nM insulin or IR overexpression. Values are means ± SE of three independent experiments, each performed in triplicate. ^a,b,c^ Mean values with dissimilar superscript letters were significantly different between each group (ANOVA post-hoc Duncan's multiple range test; P<0.05).

Insulin increased the mRNA levels of β-catenin, TCF7L2, and their target gene cyclin D1 in a dose-dependent manner, and 100 nM insulin showed protein expression levels of these genes similar to IR overexpression (P<0.05, Figure 4A). 100 nM insulin phosphorylated PI3K, AKT, and GSK3β serine residue 9. It also increased the total protein level of β-catenin, but not the alteration of TCF7L2 (Figure 4B). Moreover, nuclear translocation of β-catenin (i.e., the color green) was increased in cells treated with 100 nM insulin and INS-IR in comparison to the control (Figure 4C). This Wnt signaling activation by insulin treatment might lead to cell proliferation (MTT data) and insulin secretion (GSIS data) (P<0.05, Figures 4D and E). These results were a pattern similar to the pattern of IR overexpression (INS-IR cells) (Figure 3E).

### Transplantation of INS-IR cells in STZ-induced diabetic rats and glucose-lowering effects

**Figure 5:**
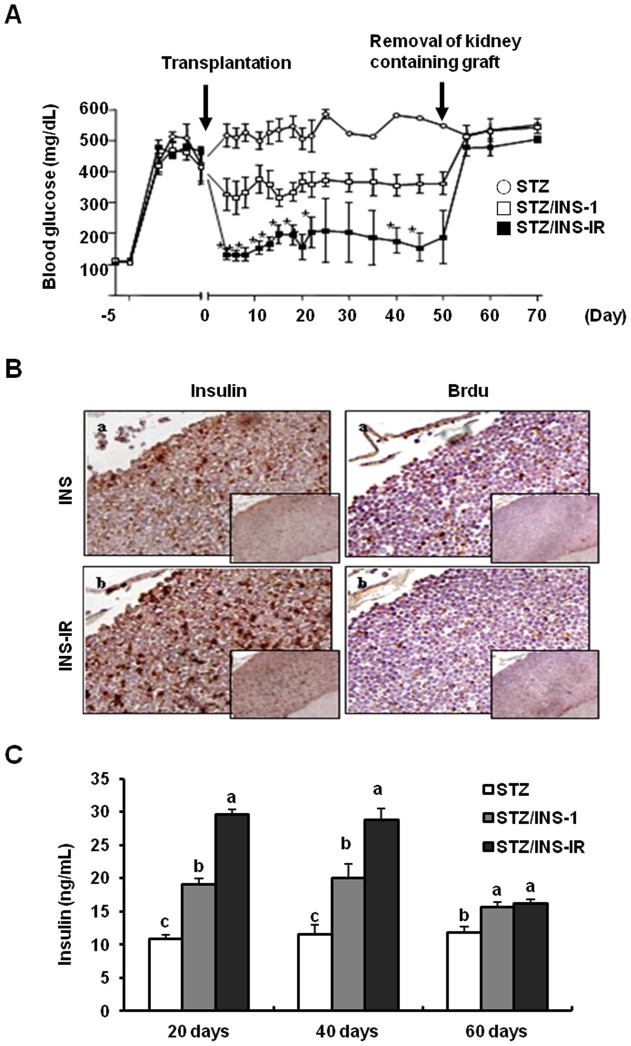
Transplantation of INS-IR cells into STZ-induced diabetic rats and the glucose-lowering effects. STZ (60 mg/kg) was injected on day 1, and 2×10^5^ INS-1 cells and INS-IR cells were implanted on day 5 (n = 5). *A*, Daily blood glucose levels before and after transplantation of INS-1 and/or INS-IR cells. *B*, Insulin expression and BrdU uptake in kidneys with transplanted INS-1 and/or INS-IR cells using immunohistochemistry; original magnification, ×100 and ×400. *C*, Serum insulin levels at 20, 40 and 60 days after transplantation of INS-1 and/or INS-IR cells. Values are mean ± SE of three independent experiments, each performed in triplicate. ^a,b,c^ Mean values with dissimilar superscript letters were significantly different between each group (ANOVA post-hoc Duncan's multiple range test; P<0.05) and *p<0.05 INS-1 vs. INS-IR (Student t-test).

INS-1 and INS-IR cells (2×10^5^) were transplanted under the kidney capsule of STZ-induced diabetic rats. Although the STZ/INS-1 group (400–500 mg/dl) showed slightly lower blood glucose levels than the STZ group (500–600 mg/dl), the STZ/INS-IR group (<200 mg/dl) showed significantly lower blood glucose levels than other groups. In addition, the level of blood glucose was maintained for 50 days. When nephrectomy was undertaken at 50 days after transplantation, the blood glucose levels climbed up sharply to higher than 400 mg/dl in both the STZ/INS-1 and STZ/INS-IR groups (P<0.05, Figure 5A). To verify whether INS-IR cells were working *in vivo*, histological examination of the transplanted cells in kidney tissues of the rats was conducted after 50 days of transplantation and serum insulin levels were determined after 20, 40 and 60 days of transplantation. The STZ/INS-IR group showed greater rates of insulin activity and proliferation (Figure 5B) than the STZ/INS-1 group. Also, The STZ/INS-IR group had higher serum insulin levels at 20 and 40 days of transplantation, but not 60 days after the removal of kidney containing graft, than the STZ/INS-1 group or STZ group (P<0.05, Figure 5C).

## Discussion

Recently, it has been shown that pancreatic β-cells are a target tissue as well as a depot of insulin. Previous studies about insulin and IR-stimulated GSIS in β-cells [17], [18] have observed that GSIS is not performed in the absence of insulin signaling [19]. Also, it has been reported that attenuated Wnt signaling perturbs pancreatic growth and TCF7L2 enhances β-cell survival and function in human pancreatic islets [20], [21]. Indeed, our study demonstrated that IR-overexpressing β-cells have higher therapeutic potential and function than pure β-cells without gene manipulation for DM and activated Wnt signaling pathway, e.g., the increase in cyclin D1 and GK or the stimulation of β-catenin nuclear translocation. These results were associated with the evidence that insulin or IR-activated insulin signaling could interplay with Wnt signaling through GSK3β phosphorylation or inducible interaction with LRP 5/6 co-receptors [9]. LRP5/6 co-receptor leads to an inactivation of GSK3β. In turn, the GSK3β can no longer phosphorylate β-catenin. This induces stabilization and nuclear translocation of β-catenin, and a consequent combination of β-catenin to TCF7L2 and transcription of the Wnt responsive genes including cyclin D1, PPARγ, and GK [12]. Based on the results of microarray-based gene expression profiling, we found the expression of LRP6 was rather lowered, but the phosphorylation of GSK3β was increased by IR overexpression, In this regard, we suggest that Wnt signaling activation by IR overexpression, occurred through GSK3β, rather than LRP5/6. Furthermore, this result is due to higher induction and activation of insulin signaling intermediates, such as PI3K and AKT, and the fast transmission of insulin signaling to Wnt signaling pathways by IR overexpression. Generally, AKT1 phosphorylates both GSK3 isotypes (GSK3α and GSK3β) on serine residues at the N terminus (serine 21 of GSK3α and serine 9 of GSK3β) [22], [23]. This phosphorylation leads to a decrease in GSK3β activity.

This interaction between insulin and Wnt signaling pathways has been recently demonstrated in a variety of organs or cells, such as kidney, bone, preadipocyte, intestinal endocrine cells, and skeletal muscle cells [8], [9], [24]–[26]. However, there is not yet known the cross-talk between insulin and Wnt signaling pathways in β-cells, and more previous studies focused on insulin repceptor substrate or IR regulation by Wnt signaling [9], [24], [27]. Furthermore, the impairment of β-cell growth and overt glucose intolerance shownin a β-cell-specific IR knockout (βIRKO) mouse [28] or an IRS-2 null mouse [29] are relevant to AKT-mediated anti-apoptotic and proliferative mechanisms [30], [31] regardless the involvement of Wnt signaling. Thus, our findings are the first report to demonstrate that the cross-talk between insulin and Wnt signaling plays central role in β-cell functionality and proliferation improvement by IR overxpression, which confirmed by the application of β-catenin, GK, and cyclin D1 siRNA to IR-overexpressing β-cell.

Indeed, better responsiveness to the glucose challenge shown in IR overexpression can be explained by GK. Commonly, GK exists as two isoforms that have independent tissue-specific promoters. In pancreatic β-cells, GK acts as a glucose sensor and contributes to the regulation of GSIS [32]. Loss of GK alleles results in permanent neonatal diabetes, whereas the heterozygous inactivating mutations of GK lead to maturity-onset diabetes of the young (MODY2) [33]. However, there are some differences between GK isoforms in the pancreas and the liver. The pancreatic GK isoform is an absolute requirement for survival, as gkdel/del (either global or β-cell-specific GK knockout mice) is lethal [34], [35]. Additionally, GK of β-cells is activated by glucose, in contrast to liver GK, which is regulated by insulin [13]. However, our collective data suggest β-catenin as a new activator for GK of β-cells in IR overexpression condition. In the presence of insulin, the protein interaction of β-catenin and PPARγ and the binding of β-catenin/PPARγ complex to PPRE in GK promoters were increased in INS-IR cells and these findings are consistent with previous studies that β-catenin coactivates PPARγ-mediated transcription on GK gene promoters in β-cells [5], [15], [36]. Also, the increased GK transcriptional activity might lead to the increase in glucose utilization, calcium mobilization, and in turn the upregulation of CREB/PDX-1/Insulin in INS-IR cells, because the increase of intracellular ATP by GK activation opens L-type Ca^2+^ channels through membrane depolarization, the increased calcium can phosphorylate the serine 133 residue of CREB through binding to its upstream gene CAMKII [37], and in turn these events increase the expression of PDX-1, a transcription factor binding to insulin gene promoter, and insulin [38].

Also, IR overexpression showed a significant increase in cell proliferation and a reduced β-cell apoptosis. At this point, it is becoming clear that IRS-2 and AKT are key signaling molecules for the maintenance of β-cell mass [39], [40]. In one study, IRS2^−/−^ mice exhibited type 2 diabetic phenotypes due to a defect in compensatory β-cell hyperplasia for hepatic insulin resistance. Furthermore, studies have shown that β-cell-specific upregulation of IRS-2 promotes β-cell growth, survival, and insulin secretion, and therefore prevents diabetes [40]. Constitutively active AKT has prevented free fatty acid-induced β-cell apoptosis via inhibition of the pro-apoptotic proteins GSK-3α/β, FoxO1, and p53 [41], [42]. In accordance with previous observations, our results also demonstrate that amplification of insulin signaling through hIR reduces IRS-2 serine/threonine phosphorylation (inactive form), and by contrast, increases AKT serine phosphorylation (active form) and decreases the rate of β-cell apoptosis. More interestingly, both β-catenin and its target gene cyclin D1 knockdown accelerated glucose-induced apoptosis up to the similar rate of apoptosis shown in AKT and IRS-2 gene depletion. These results are consistent with another study [43] which showed that purified WNT3a (which is known to activate the canonical WNT pathway) stimulates proliferation of both the mouse β-cell line (MIN6) and primary mouse pancreatic β-cells, possibly through cell cycle regulator cyclin D1, and 3-month-old rat insulin I promoter (RIP)-Cre, β-catenin ^active^ mice showed a threefold increase in the production of Ki67 in pancreatic β-cells. Taken together, these observations suggest that β-catenin/cyclin D1 is as necessary for proliferation as IRS-2 and/or AKT in IR-overexpressing β-cells. In addition, the activation of Akt is one of the most frequent alterations observed in human cancer and tumor cells due to its characteristic of AKT for survival. Indeed, AKT2 is amplified and overexpressed in pancreatic carcinomas and AKT2 antisense RNA can greatly diminish the tumorigenic phenotype of pancreatic cancer cells harboring amplified AKT2 [44]. Considering this point, constitutively active AKT is not appropriate for clinical applications. Also, it has been recently demonstrated that autocrine insulin signaling is essential to up-regulate both basal and glucose-stimulated PIP3 levels, and this result is completely abrogated by IR blockade. The binding of PIP3 to the PH domain of AKT anchors Akt to the plasma membrane and allows to the plasma membrane and allows its phosphorylation and activation by PDK1 (Phosphoinositide-Dependent Kinase-1) [45]. In this regard, the manipulation of IR gene of PI3K/AKT upstream would have better beneficial effect than AKT in β-cells transplantation for DM patients. However, IR-enhanced proliferation of insulinoma cells has the possibility not to be replicated in endogenous β-cells because the cell cycle in insulinoma cells is different from primary β-cells.

These positive results, however, are limited to studies *in vitro*. Thus, we transplanted the same number of INS-1 cells and INS-IR cells (cells with IR overexpression) to STZ-induced diabetic rats for testing therapeutic efficiency. There was a striking difference in the glucose control between the two groups. Although the blood glucose levels of the STZ/INS-1 group were still high (400–500 mg/dl) after transplantation, the STZ/INS-IR group displayed near-normal blood glucose level for up to 50 days and increased serum insulin levels during transplantation.

In conclusion, our study seems to be the first one to demonstrate that the cross-talk between insulin and Wnt signaling operates IR-overexpressing β-cell functionality and proliferation improvement through GK and/or cyclin D1-dependent Wnt pathways. In addition, this study proved the higher therapeutic efficiency of IR-overexpressing β-cells than pure β-cells. In this regard, transplantation of IR-overexpressing β-cells will be a promising potential tool for the treatment of insulin-dependent DM rather than insulin drug, islet transplantation, xenotransplantation and stem cell technology because these therapeutic approaches have their own limitations, such as weight gain, hypoglycemia, the limited donor supply, immune rejection, tumor formation, and ethical issues [46].

## Supporting Information

File S1 **Figure S1, Clone 4 selection as a primary clone, based on GSIS.** (A), Four clones were further screened for the positive clones through glucose-stimulated insulin secretion response. Based on insulin secretion response (Clone 4 responded the best to 16.7 or 33.3 mM glucose stimulus), Clone 4 was selected as the primary clone for INS-IR cell. All the further studies were performed on the primary clone, Clone 4. Data are expressed as mean ± SEM (n = 3–5). (B), the process of establish stable cells. ^a,b,c^Mean values with dissimilar superscript letters were significantly different between three groups (ANOVA post hoc Duncan's multiple range test; P<0.05). **Figure S2, Glucose uptake, intracellular ATP content and insulin release in response to 16.7 mM glucose challenge.** 16.7 mM glucose did not increase glucose uptake (A), intracellular ATP content (B), and first-phase insulin release (C) in INS-IR cells as compared with INS-1 cells. Data are expressed as mean ± SEM (n = 3–5 ^†^ represented significant differences between two groups (Student's t-test; P<0.05). **Figure S3, The magnitude of β-catenin knockdown obtained in β-catenin siRNA.** INS-IR cells were transfected with β-catenin siRNA for 48h. RT-PCR was performed to confirm β-catenin knockdown. GAPDH was used as the housekeeping gene. RT-PCR bands were quantified by using Image J program and then corrected by GAPDH. Data are expressed as mean ± SEM (n = 3–5). ^*^represented significant differences between two groups (Student's t-test; P<0.05). **Figure S4, The magnitude of GK knockdown obtained in GK siRNA.** INS-IR cells were transfected with GK siRNA for 48 h. RT-PCR was performed to confirm GK knockdown. β-actin was used as the housekeeping gene. RT-PCR bands were quantified by using Image J program and then corrected by β-actin. Data are expressed as mean ± SEM (n = 3–5). ^*^represented significant differences between two groups (Student's t-test; P<0.05). **Figure S5, The magnitude of knockdown obtained in AKT1, IRS-2, β-catenin, and cyclin D1 siRNA.** INS-1 or INS-IR cells were transfected with AKT1, IRS-2, β-catenin, and cyclin D1 siRNA for 48 h. RT-PCR was performed to confirm each gene knockdown. GAPDH or β-actin were used as the housekeeping gene. RT-PCR bands were quantified by using Image J program and then corrected by GAPDH or β-actin. Data are expressed as mean ± SEM (n = 3–5). ^*^represented significant differences between two groups (Student's t-test; P<0.05). **Figure S6, The magnitude of knockdown obtained in AKT1, IRS-2, β-catenin, and cyclin D1 siRNA by Western blot.** INS-IR cells were transfected with AKT1, IRS-2, β-catenin, and cyclin D1 siRNA for 48 h. Western blot was performed to confirm each gene knockdown. β-actin were used as the housekeeping gene. Western bands were quantified by using Image J program and then corrected by β-actin. Data are expressed as mean ± SEM (n = 3–5). ^*^represented significant differences between two groups (Student's t-test; P<0.05).
(DOC)
