# Supplementary material for: Insulin Receptor-Overexpressing β-Cells Ameliorate Hyperglycemia in Diabetic Rats through Wnt Signaling Activation
Source: PLoS One. 2013 Jul 9;8(7):e67802. doi: 10.1371/journal.pone.0067802 (PMC3706479; doi:10.1371/journal.pone.0067802)
Supplement: File S1 — Figure S1, Clone 4 selection as a primary clone, based on GSIS. (A), Four clones were further screened for the positive clones through glucose-stimulated insulin secretion response. Based on insulin secretion response (Clone 4 responded the best to 16.7 or 33.3 mM glucose stimulus), Clone 4 was selected as the primary clone for INS-IR cell. All the further studies were performed on the primary clone, Clone 4. Data are expressed as mean ± SEM (n = 3–5). (B), the process of establish stable cells. a,b,cMean values with dissimilar superscript letters were significantly different between three groups (ANOVA post hoc Duncan's multiple range test; P<0.05). Figure S2, Glucose uptake, intracellular ATP content and insulin release in response to 16.7 mM glucose challenge. 16.7 mM glucose did not increase glucose uptake (A), intracellular ATP content (B), and first-phase insulin release (C) in INS-IR cells as compared with INS-1 cells. Data are expressed as mean ± SEM (n = 3–5 † represented significant differences between two groups (Student's t-test; P<0.05). Figure S3, The magnitude of β-catenin knockdown obtained in β-catenin siRNA. INS-IR cells were transfected with β-catenin siRNA for 48h. RT-PCR was performed to confirm β-catenin knockdown. GAPDH was used as the housekeeping gene. RT-PCR bands were quantified by using Image J program and then corrected by GAPDH. Data are expressed as mean ± SEM (n = 3–5). *represented significant differences between two groups (Student's t-test; P<0.05). Figure S4, The magnitude of GK knockdown obtained in GK siRNA. INS-IR cells were transfected with GK siRNA for 48 h. RT-PCR was performed to confirm GK knockdown. β-actin was used as the housekeeping gene. RT-PCR bands were quantified by using Image J program and then corrected by β-actin. Data are expressed as mean ± SEM (n = 3–5). *represented significant differences between two groups (Student's t-test; P<0.05). Figure S5, The magnitude of knockdown obtained in AKT1, IRS-2, β-catenin [file pone.0067802.s001.doc]

**Supplementary Data**

**Insulin Receptor-Overexpressing β-Cells Ameliorate Hyperglycemia in Diabetic Rats through Wnt Signaling Activation**

Mi-Hyun Kim, Seung-Hyun Hong, Moon-Kyu Lee

**Correspondence: Moon-Kyu Lee, MD, PhD.** Division of Endocrinology and Metabolism, Department of Medicine Samsung Medical Center, Sungkyunkwan University School of Medicine, Ilwon-dong 50, Gangnam-gu, 135-710, Seoul, Korea. Phone: +82-2-3410-3431 Fax: +82-2-3410-0393 E-mail: [leemk@skku.edu](mailto:leemk@skku.edu)

**
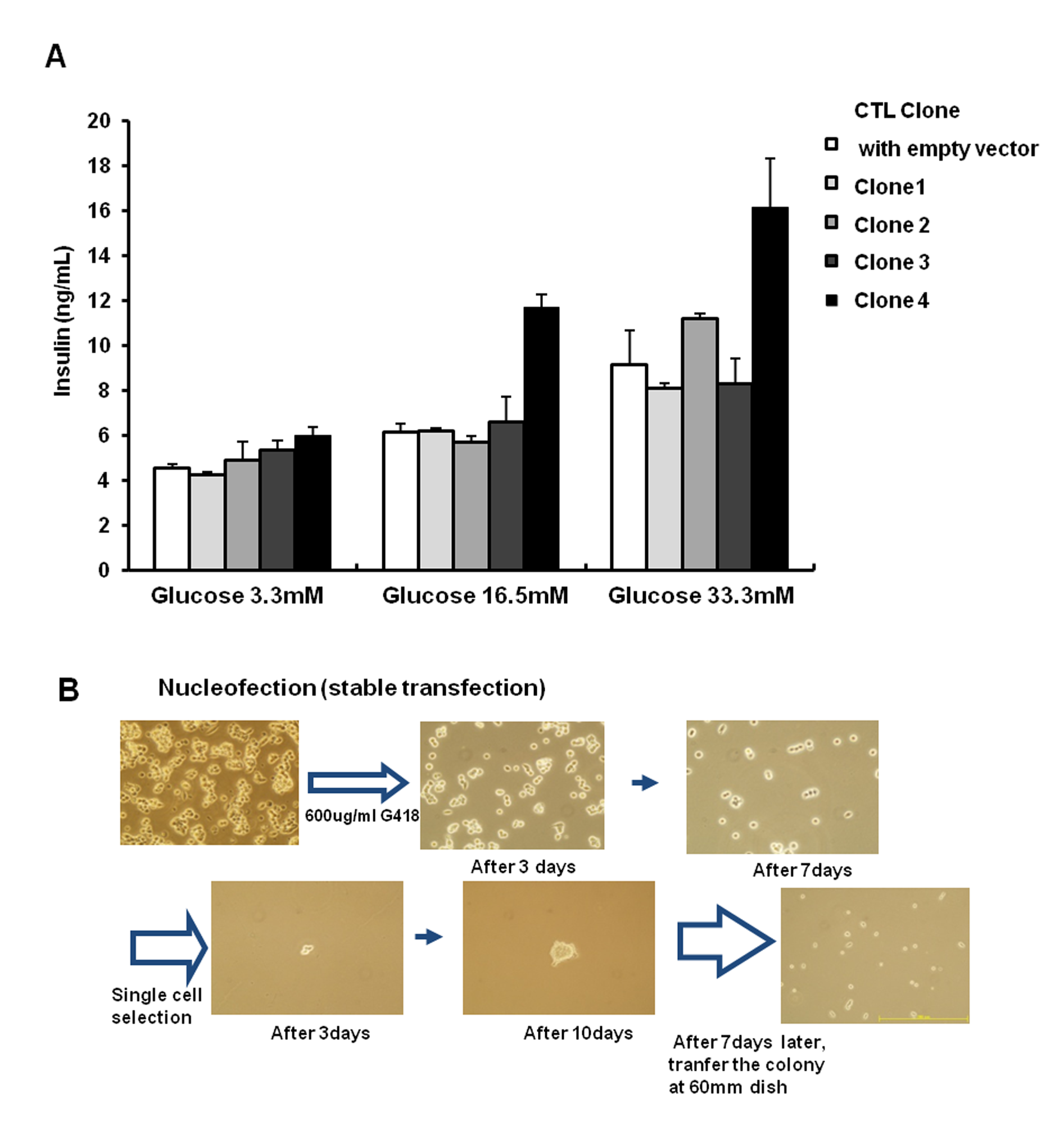
**

**FIGURE S1, A and B**

**
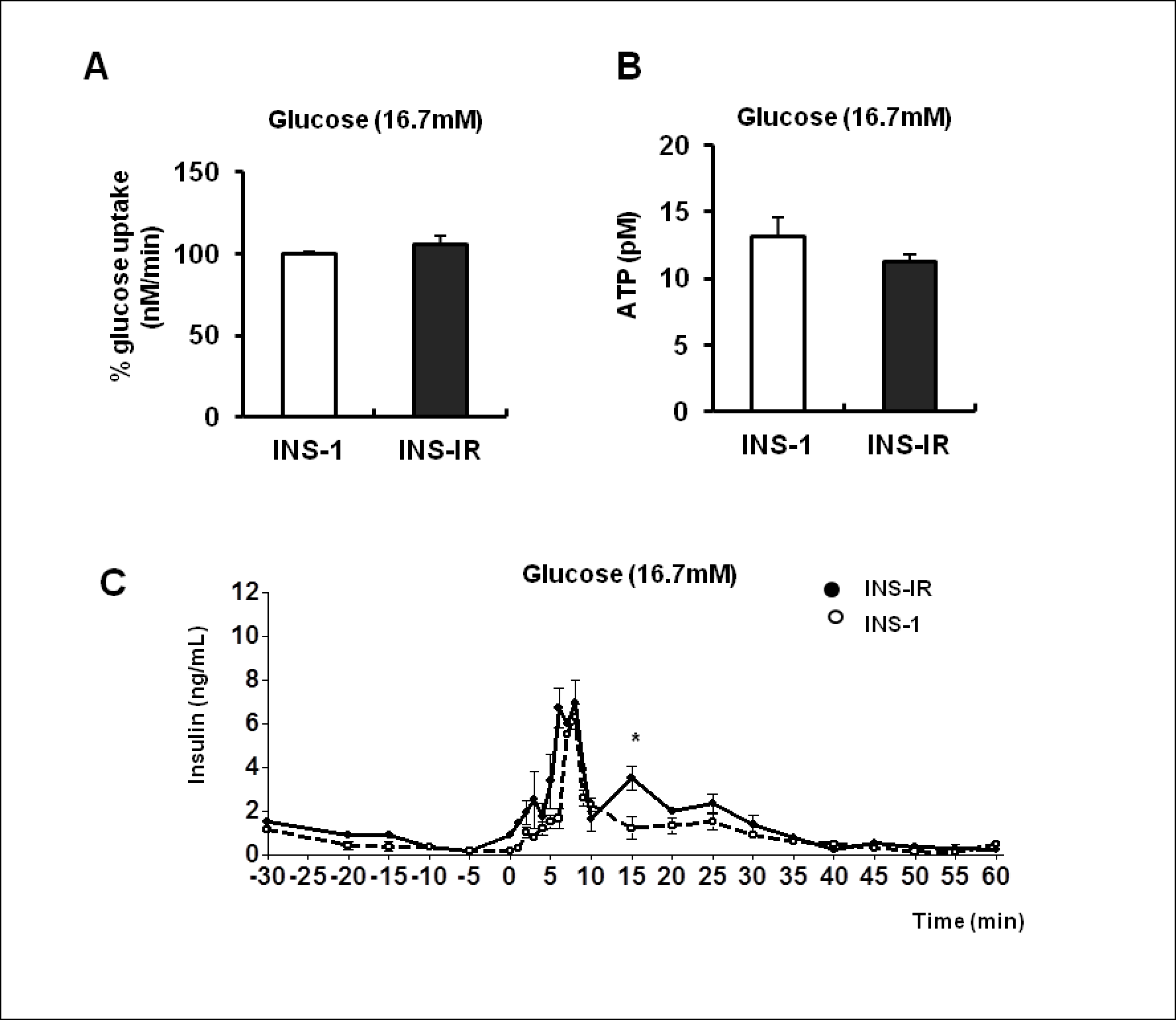
**

**FIGURE S2**


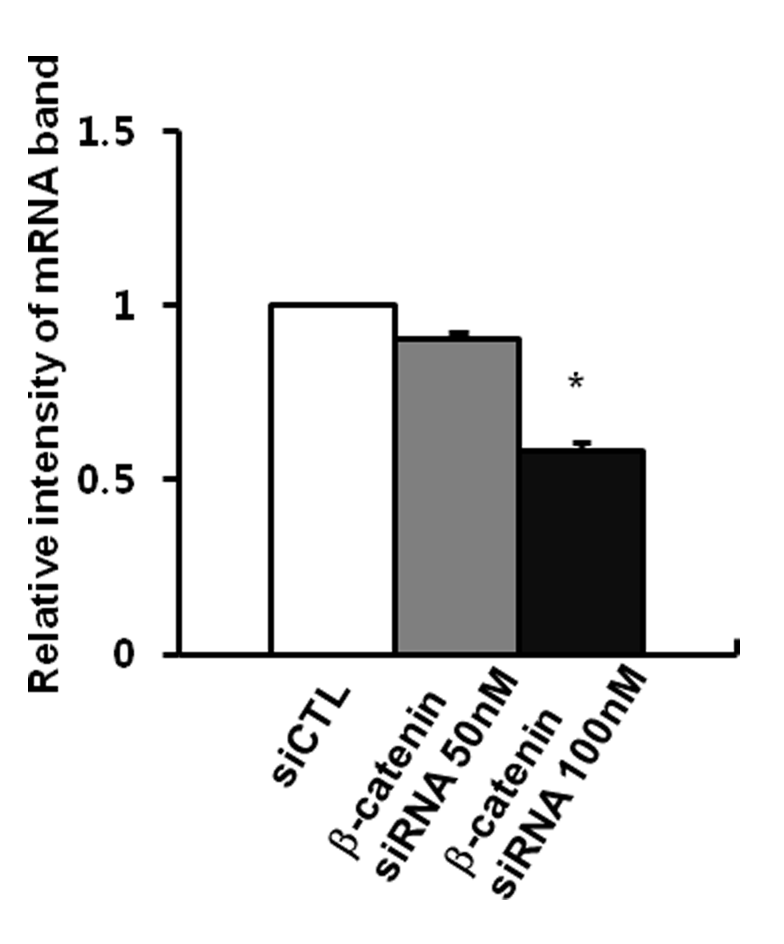


**FIGURE S3**

**
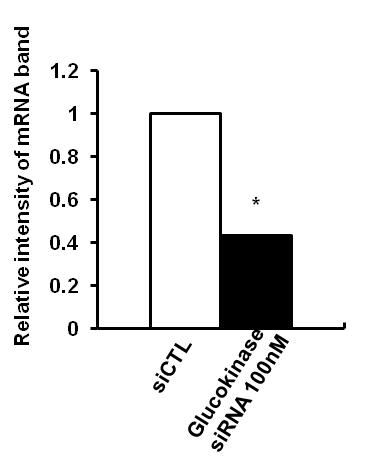
**

**FIGURE S4**


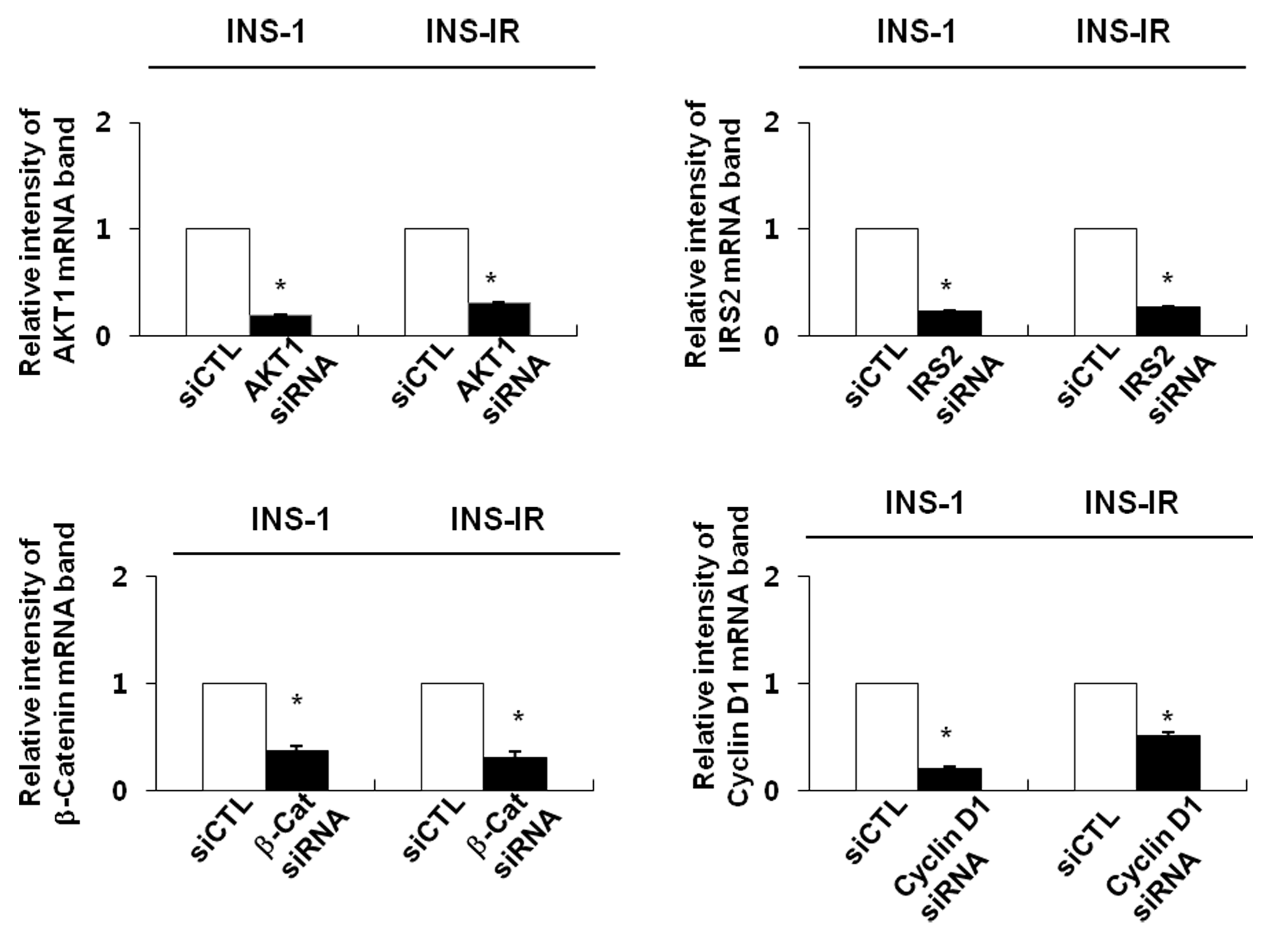


**FIGURE S5**

**
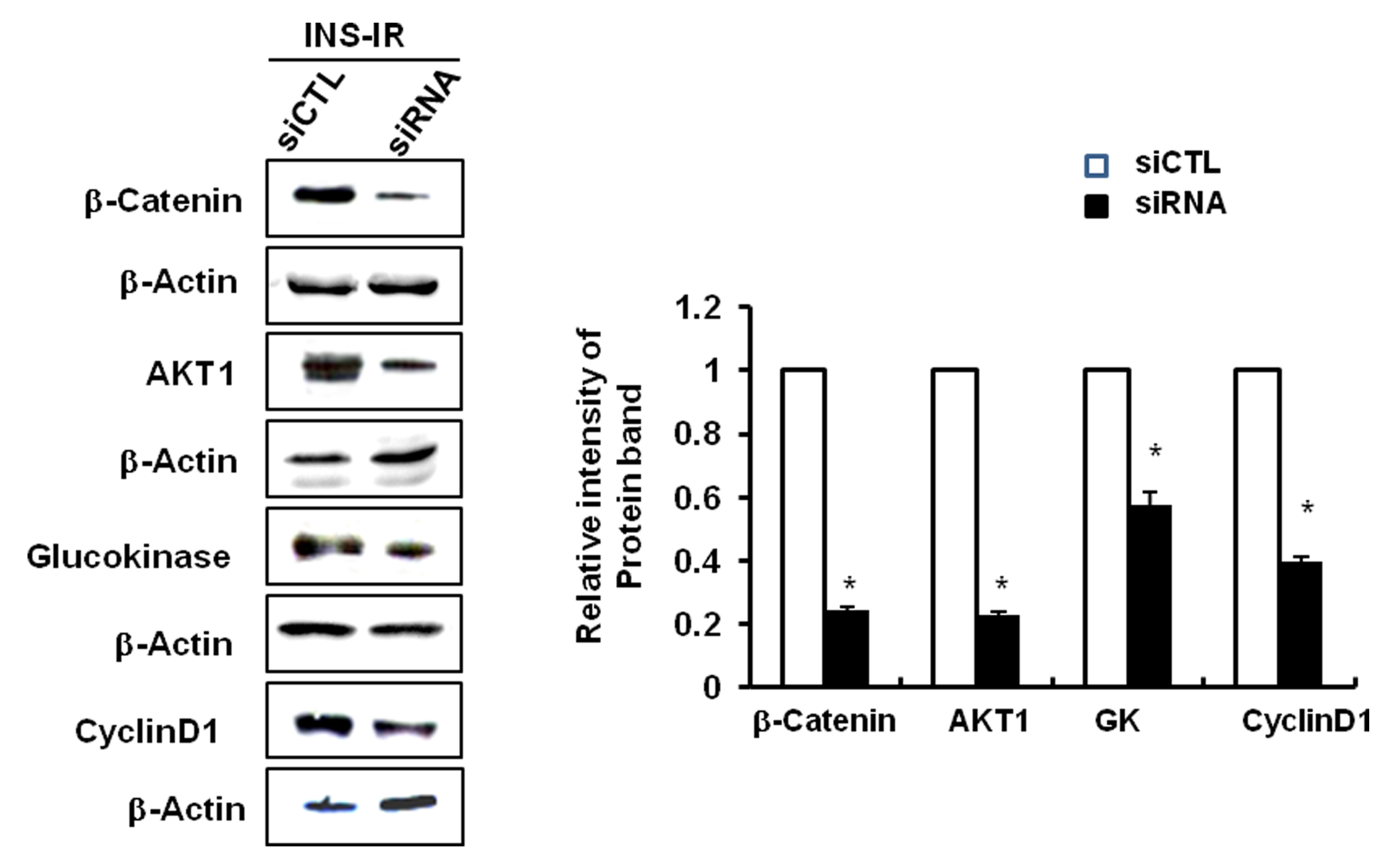
**

**FIGURE S6**
